# Evaluating the anti *Mycobacterium tuberculosis* activity of *Alpinia galanga* (L.) Willd. axenically under reducing oxygen conditions and in intracellular assays

**DOI:** 10.1186/1472-6882-14-84

**Published:** 2014-03-04

**Authors:** Pooja Gupta, Purva Bhatter, Desiree D’souza, Monica Tolani, Poonam Daswani, Pundarikakshudu Tetali, Tannaz Birdi

**Affiliations:** 1 The Foundation for Medical Research, 84-A, R.G. Thadani Marg, Worli, Mumbai 400018, Maharashtra, India; 2 Naoroji Godrej Centre for Plant Research, Lawkim Motors Group Campus, Shindewadi, Shirwal, Satara, Maharashtra 412801, India

**Keywords:** *Mycobacterium tuberculosis*, Medicinal plants, *Alpinia galanga*, Anaerobic assay, Intracellular assays

## Abstract

**Background:**

In tuberculosis (TB), the steadily increasing bacterial resistance to existing drugs and latent TB continue to be major concerns. A combination of conventional drugs and plant derived therapeutics can serve to expand the antimicrobial spectrum, prevent the emergence of drug resistant mutants and minimize toxicity. *Alpinia galanga*, used in various traditional medicines, possesses broad spectrum antibacterial properties. The study was undertaken to assess the antimycobacterial potential of *A. galanga* in axenic (under aerobic and anaerobic conditions) and intracellular assays.

**Methods:**

Phytochemical analysis was done using HPTLC. The acetone, aqueous and ethanolic extracts (1, 10, 25, 50 and 100 μg/ml) of *A. galanga* were tested axenically using Microplate Alamar Blue Assay (MABA) against *Mycobacterium tuberculosis* (M.tb) H37Rv and three drug sensitive and three multi drug resistant clinical isolates. The activity of the extracts was also evaluated intracellularly in A549 cell line against these strains. The extracts active under intracellular conditions were further tested in an axenic setup under reducing oxygen concentrations using only H37Rv.

**Results:**

1´ acetoxychavicol acetate, the reference standard used, was present in all the three extracts. The acetone and ethanolic extracts were active in axenic (aerobic and anaerobic) and intracellular assays. The aqueous extract did not demonstrate activity under the defined assay parameters.

**Conclusion:**

*A. galanga* exhibits anti M.tb activity with multiple modes of action. Since the activity of the extracts was observed under reducing oxygen concentrations, it may be effective in treating the dormant and non-replicating bacteria of latent TB. Though the hypothesis needs further testing, *A. galanga* being a regular dietary component may be utilized in combination with the conventional TB therapy for enhanced efficacy.

## Background

Tuberculosis (TB) is an infectious bacterial disease caused by *Mycobacterium tuberculosis* (M.tb). It commonly affects the lungs but may also affect other parts of the body viz., the brain, spine and the kidneys. Not every individual infected with M.tb presents with symptoms, referred to as latent TB (LTBI). As a result, two TB-related conditions exist: active TB disease and latent TB infection (LTBI) [1]. Whilst individuals with LTBI are asymptomatic and non infectious, they are at a risk of progression to active disease. In LTBI, due to quantitative metabolic shutdown, the dormant bacilli fail to respond to drug therapies which target multiplying bacteria. Thus identification and treatment of LTBI is equally important to ensure complete elimination of TB.

The steadily increasing bacterial resistance to existing drugs is a serious problem [2,3], resulting in the urgent need for development of new TB drugs and shorter treatment regimens. This has led to the search for new classes of antimicrobial agents. Unlike synthetic drugs, antimicrobials of plant origin are found to exhibit fewer side effects and have the therapeutic potential to treat many infectious diseases [4,5]. Development of easier, rapid and safer screening techniques has intensified the search of chemical entities from botanicals and other natural resources for activity against Mycobacteria species [6]. *In vitro* inhibitory activity of crude extracts and/or pure active compounds extracted from plants against M.tb and its related species has been extensively reported [7-10].

*Alpinia galangal* (L.) Willd., family Zingiberaceae commonly referred to as galangal, is widely cultivated in South-east Asian countries such as Philippines, Indonesia, Thailand, India, and China [11]. It is extensively used in diets as well as in the traditional systems of medicine viz., Thai, Ayurveda, Unani and Chinese folk medicine [12]. Galangal has been known for its use as anti-inflammatory, antipyretic, emmenagogue, carminative, abortifacient and aphrodisiac and is used in the treatment of various diseases such as renal calculus, diabetes, heart diseases, bronchitis, rheumatism, chronic enteritis and kidney disorders [12,13]. Among other components, it is reported to contain tannins, glycosides, essential oils, phenol, carbohydrates and monoterpenes. Antimicrobial activity of galangal [13] and the *Alpinia* species [14] has also been reported earlier. Additionally 1´ acetoxychavicol acetate, a phenylpropanoid, isolated from *A. galanga* and *A. nigra* is specifically known to possess antituberculous activity [14]. Crude extract of *A. galanga* has been demonstrated to have an activity similar to that of isoniazid [15]. However, Soundhari and Rajarajan [16] have demonstrated the activity of galangal in isoniazid resistant clinical strains *in-vitro*. The possible reason for this discrepancy needs to be elucidated. Phongpaichit et al. evaluated the antimycobacterial activity of extracts from galangal and suggested its use as self-medication for treatment of TB in AIDS patients in Thailand [14]. Although there are reports of activity of *A. galanga* on axenic aerobic growth of M.tb, to our knowledge, there are no studies reporting its activity against M.tb under intracellular conditions and reduced oxygen concentrations. It is important that the antimycobacterial activity of plants be measured under hypoxic conditions since it is a model for non replicating and dormant bacilli. Besides, the intracellular environment in which the TB bacterium resides is anaerobic and is characterized by the switch from aerobic/microaerophilic to anaerobic respiratory pathways by utilisation of lipids as a carbon source [17].

## Methods

### Plant material

*Alpinia galanga* was selected for the present study on the basis of its broad antibacterial properties [11,12]. The plant was sourced from Kerala Agricultural University and cultivated at Naoroji Godrej Centre for Plant Research (NGCPR). The plant material was authenticated by Dr. P. Tetali, a taxonomist at NGCPR. A voucher specimen of the plant has been deposited at Botanical Survey of India (BSI), Western Center, Pune, India, under the herbarium number 131745.

### Extract preparation

Coarsely powdered plant material (rhizomes) was sequentially extracted [18] with acetone, ethanol and distilled water using the Soxhlet apparatus. 300 ml of respective solvent was continuously refluxed with 25 g of plant material for a period of 24-30 hours for efficient extraction of the phytoconstituents. Post ethanol extraction and evaporation of the solvent, the aqueous extract was prepared by boiling the plant material until the volume of water was reduced to 25%. The aqueous extract was lyophilized (Thermo Fisher Scientific, USA) and the acetone and ethanolic extracts were allowed to air dry. The percent yields of the acetone, aqueous and ethanolic extracts were 2.92, 23.6 and 6.84 (w/w) respectively. For the assays, the extracts were reconstituted at 20 mg/ml concentration in dimethyl sulfoxide (DMSO, SD fine Chemicals, India), filtered through 0.2 μm, 25 mm DMSO resistant Acrodisc syringe filters (Pall Corporation, USA) and stored at -20°C for up to 2 weeks.

### Phytochemistry

The extracts of *A. galanga* were subjected to phytochemical fingerprinting using High Performance Thin Layer chromatography (HPTLC). The extracts were spotted on pre-coated Silica gel G60 F254 TLC plates (Merck, Germany) along with the reference standard viz., 1´ acetoxychavicol acetate (Natural Remedies, Bangalore, India) using Linomat V Automatic Sample Spotter (CAMAG, Switzerland), run in a ‘twin trough TLC chamber’, dried and visualized in ‘CAMAG TLC visualizer’ pre and post derivatization with anisaldehyde-sulpuric acid. The mobile phase used was Toluene: Acetone (7:3).

### Bacterial culture

The reference M.tb laboratory strain H37Rv, susceptible to the first line drugs, along with three drug susceptible and three multi drug resistant clinical isolates [3] were used for MABA and intracellular assay. The details of the isolates used for the study are presented in Table 1.

**Table 1:** **Characteristics of strains used for testing the antimycobacterial activity of *A*. *galanga***

**Sr. no**	**Strain ID**	Drug susceptibility profile*	***rpoβ***^***Ψ ***^***genotype***	***katG***^***Ψ ***^***genotype***	**inhA promoter genotype**
1	H37Rv	Susceptible	Wild type	Wild type	Wild type
2	S1	Susceptible	Wild type	Wild type	Wild type
3	S2	Susceptible	Wild type	Wild type	Wild type
4	S3	Susceptible	Wild type	Wild type	Wild type
5	R1	Resistant to HR	D516V mixed	S315T1/T2	A16G mixed
6	R2	Resistant to HERZ	D516V mixed	S315T1/T2	A16G mixed
7	R3	Resistant to HERZ	S531L	S315T1/T2 mixed	Wild type

Antimycobacterial activity of *A.galanga* under anaerobic conditions was tested using only H37Rv.

H-isoniazid, E-Ethambutol, R-Rifampicin, Z-Pyrazinamide.

S- Drug susceptible strain.

R- Drug resistant strain.

*Phenotypic drug resistance was ascertained using the BACTEC MGIT 960 system.

^*Ψ*^Genotypic drug resistance was ascertained using the GenoType MTBDR*plus* assay (Hain Lifescience, Germany). Mixed indicates heteroresistance (presence of wild type and mutant bands).

### MABA

The assay was performed as reported by Collins and Franzblau [19] and Webster et al. [20]. Briefly, 100 μl of 0.5 × 10^6^/ml of the M.tb strains (Viable M.tb, VMTB) were cultured in 7H9 medium (supplemented with ADC and 0.5% glycerol) in the presence of the plant extracts (1, 10, 25, 50 and 100 μg/ml) in a Nunc™ flat bottom 96 well plate (Nunclon, Denmark). The controls maintained for all the tested strains included: medium, DMSO (at a volume that is used for the highest concentration of plant extract), 1:100 VMTB and 2 μg/ml Rifampicin (RIF) (Sigma-Aldrich, USA). To check the interaction of the plant extracts with Alamar Blue, additionally wells with plant extracts and media were also maintained. The plates were incubated at 37°C for seven days. Post incubation, 10 μl of Alamar Blue dye (Invitrogen, USA) [5% (v/v)] diluted 1:1 in 7H9 medium (supplemented with ADC and 0.5% glycerol) was added and the plates were reincubated for 30 hours. The Optical Density (O.D.) of the wells was measured at 600 nm and 570 nm in an ELISA reader (Thermo Fisher Scientific, USA), and the percent reduction of Alamar Blue dye was calculated as per the manufacturer’s instructions. Use of percent reduction to screen plant extracts allowed identification of those extracts with marginal activity (not resulting in 99% kill). Triplicate wells were maintained for each variable in every assay and all the assays were performed thrice and the data was analyzed as Mean ± SD.

The results were interpreted based on the percent reduction of the dye which is directly proportional to the bacterial growth. The extracts were considered to be active if the percent reduction value of Alamar Blue dye was less than that observed for the 1:100 VMTB control [21].

Internal Quality Control was performed by comparing the results of MABA to the Bactec MGIT960 system (BD, USA). As the aqueous extract was found to be ineffective, only acetone and ethanolic extracts of *A. galanga* were tested against H37Rv and the six clinical isolates.

### Intracellular assay

The human lung carcinoma epithelial cell line, A549 (NCCS, Pune, India), was grown in DMEM (GibcoBRL, UK) supplemented with 10% fetal calf serum (FCS) and 4 μg/ml of gentamycin. 10^6^ cells/well were seeded in a 96 well plate and were infected with H37Rv, drug susceptible and drug resistant strains at a multiplicity of infection (MOI) of 1:1 for 6 hrs. Post infection, the cells were treated with amikacin [22] (standardized to 50 μg/ml) for 2 hrs to kill the remaining extracellular bacteria. The excess amikacin was washed off and the infected cells were incubated with the plant extracts at a concentration of 25 μg/ml and 100 μg/ml overnight in DMEM with 5% FCS. On the following day the plant extracts were washed off and the cells maintained in DMEM with 3% FCS. The gradual reduction in FCS concentration ensured that the cell line did not over grow. On 0, 3rd, 5th, 7th and 10th day, post infection, the cells were lysed with 0.1% sodium dodecyl sulphate (SDS) to release the intracellular bacteria. The lysate was ten fold serially diluted with PBS and 10 μl of the two highest dilutions were spotted onto Middlebrook 7H11 (MB7H11) agar plates supplemented with OADC (Becton Dickinson, USA) and 0.5% glycerol. The plates were incubated at 37°C for three weeks and the Colony Forming Units (CFUs) enumerated.

### Double stimulus

The intracellular assays were repeated using the above mentioned protocol with the following modification. The infected cells were given a second stimulus of the plant extracts (25 μg/ml) on the fifth day post infection. This was done to evaluate if a double stimulus of the plant extracts would augment the intracellular killing of the bacteria.

The inhibition of bacterial growth was represented as percent inhibition calculated using the following formula:

$$\begin{array}{l} \%\mathrm{inhibition}\;=\;\left(\left(\mathrm{growth}\;\mathrm{under}\;\mathrm{control}\;\mathrm{conditions}\right)\right)\\ \;\;-\mathrm{growth}\;\mathrm{under}\;\mathrm{experimental}\\ \;\;\;\left(\mathrm{conditions}\right)/\mathrm{growth}\;\mathrm{under}\;\\ \;\;\;\left(\mathrm{control}\;\mathrm{conditions}\right)*100 \end{array}$$

### Axenic assay under differential oxygen concentration

The assay was carried out using only the reference M.tb laboratory strain H37Rv, susceptible to the first line drugs.

Ten ml per tube of Middlebrook 7H9 broth, supplemented with ADC and 0.5% glycerol, was aliquoted and bacterial suspension containing 10^4^ CFU/ml was inoculated into the tubes. Since significant intracellular activity was observed with higher concentration of plant extracts, 100 μg/ml of plant extract was used as the final concentration per tube. Positive and negative growth controls along with a rifampicin (1 μg/ml) control were maintained. The above set up in triplicate was subjected to differentially reducing oxygen concentration viz., aerobic, microaerophilic and anaerobic conditions. The microaerophilic conditions were obtained using the candle jar method [23]. An anaerobic jar with a gas pack (HiMedia, India) was used to create anaerobic conditions. The change in colour of the indicator tablets, provided by the manufacturer, from pink to purple was indicative of anaerobic conditions. To ensure that the bacterium had completed sufficient number of replication cycles, the sets were incubated for a period of 10 days. Post incubation each of the tubes were vortex mixed, serially diluted tenfold and 10 μl of this dilution was spotted on MB7H11 agar plate supplemented with OADC and 0.5% glycerol to enumerate the CFUs.

## Results

### Phytochemical fingerprinting

**Figure 1:**
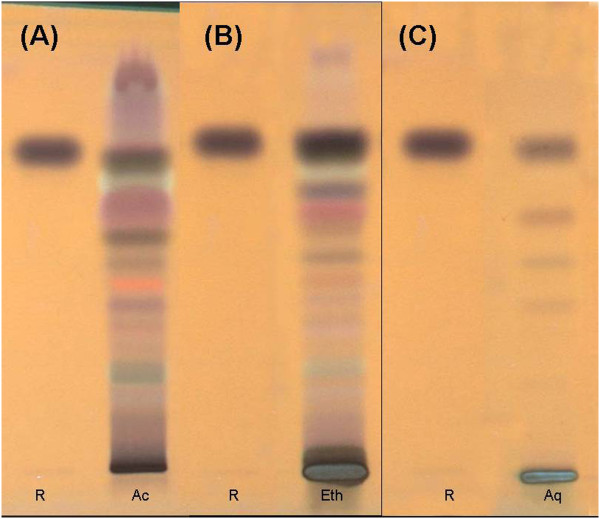
**HPTLC fingerprinting of the extracts of *Alpinia galanga*.** Profile of acetone extract **(A)**; ethanolic extract **(B)**; aqueous extract **(C)**; scanned at 366 nm post derivatization with anisaldehyde – sulphuric acid. R – Reference compound 1´ acetoxychavicol acetate, Ac – Acetone extract, Eth – Ethanolic extract, Aq – Aqueous extract.

The chromatogram of the HPTLC fingerprinting of the three extracts scanned at 366 nm post derivatization with anisaldehyde – sulphuric acid has been presented in Figure 1. As can be seen from this Figure 1’ acetoxychavicol acetate, the reference standard used, was found to be present in all the extracts with an R_f_ value of 0.67.

### MABA

The antibiotic susceptibility profile of the isolates performed using MABA was in concordance with the MGIT960 assay.

It was observed that the lower concentrations (1 and 10 μg/ml) of the three *A. galanga* extracts (acetone, aqueous and ethanolic) did not exhibit any antibacterial activity. However significant activity was observed at the higher concentrations (25, 50 and 100 μg/ml). The acetone and ethanolic extracts were found to be the most effective against all the isolates (Table 2) unlike the aqueous extract which did not show any significant activity. The higher concentration (100 μg/ml) of the extracts showed increased inhibition of all the strains.

**Table 2:** **Antimycobacterial activity of different extracts of *A. galanga *** **using microplate alamar blue assay**

**Strains**	**1: 100 VMTB**	***VMTB***	***A. galanga *** **extracts**					
			**Acetone (μg/ml)**			**Ethanolic (μg/ml)**		
			**25**	**50**	**100**	**25**	**50**	**100**
% reduction of Alamar Blue dye								
H37Rv	21.71 ± 2.84	66.05 ± 5.03	56.76 ± 9.51	25.10 ± 11.15	**18.51 ± 3.84**	57.27 ± 6.11	**14.13 ± 6.46**	**12.88 ± 7.10**
S1	21.09 ± 3.42	56.27 ± 2.30	53.84 ± 2.47	48.93 ± 11.10	**21.16 ± 6.22**	47.75 ± 13.04	35.60 ± 2.62	**16.63 ± 4.40**
S2	22.33 ± 7.00	59.17 ± 4.93	52.78 ± 6.74	42.90 ± 12.23	**15.23 ± 6.17**	41.45 ± 5.70	**14.31 ± 6.64**	**14.87 ± 7.91**
S3	19.83 ± 1.80	59.74 ± 10.58	55.58 ± 17.71	52.89 ± 16.92	**18.21 ± 2.29**	42.05 ± 6.60	32.15 ± 7.40	**18.55 ± 4.40**
R1	14.40 ± 5.71	51.08 ± 11.91	34.01 ± 6.32	25.57 ± 6.85	**21.24 ± 5.62**	23.39 ± 3.53	**16.89 ± 1.10**	**13.08 ± 5.78**
R2	22.11 ± 2.47	60.93 ± 9.00	53.38 ± 8.37	36.98 ± 8.84	**20.04 ± 1.22**	36.49 ± 4.06	**19.74 ± 4.38**	**15.74 ± 3.86**
R3	16.73 ± 4.14	50.50 ± 4.04	37.10 ± 5.03	30.48 ± 8.23	**13.95 ± 2.06**	42.59 ± 7.22	**14.25 ± 3.88**	**12.25 ± 2.87**

Values in bold indicate those parameters for which the percent reduction in plant extract containing wells was less than that for the 1:100 VMTB control.

The acetone and ethanolic extract of *A. galanga* were also tested against H37Rv and the clinical isolates in the MGIT960 system. The activity recorded was concordant with the MABA results.

### Intracellular assays

The preliminary results of the intracellular assay performed at a concentration of 25 μg/ml showed that the aqueous extract was ineffective against all the strains tested. The acetone extract was the most efficacious with more than 80% inhibition against 5/7 isolates (Table 3). The ethanolic extract showed more than 80% inhibition against 4/7 isolates (Table 3).

**Table 3:** Percent inhibition of bacterial growth using intracellular (A549) assays

**Sr. no.**	**Strains**	***A. galanga *** **extracts**					
		**Acetone (μg/ml)**			**Ethanolic (μg/ml)**		
		**25**		**100**	**25**		**100**
		**Single stimulus**	**Double stimulus**		**Single stimulus**	**Double stimulus**	
1	H37Rv	78.25 ± 6.14	94.58 ± 2.98	98.06 ± 0.24	78.41 ± 5.00	98.06 ± 0.24	98.06 ± 0.24
2	S1	83.34 ± 7.76	99.42 ± 0.50	99.37 ± 0.05	86.20 ± 12.04	98.88 ± 1.92	99.37 ± 0.05
3	S2	75.62 ± 2.23	94.47 ± 3.88	99.21 ± 0.02	73.09 ± 3.41	98.86 ± 0.44	99.21 ± 0.02
4	S3	96.41 ± 0.76	97.69 ± 1.25	95.18 ± 1.51	83.11 ± 2.33	100 ± 0	95.20 ± 1.82
5	R1	86.70 ± 5.11	95.00 ± 4.69	94.60 ± 1.30	82.21 ± 12.17	90.89 ± 6.43	97.74 ± 1.11
6	R2	89.33 ± 2.67	98.00 ± 0.00	93.56 ± 2.48	85.33 ± 5.81	93.78 ± 0.20	99.08 ± 0.52
7	R3	91.11 ± 1.92	94.36 ± 2.67	100 ± 0	57.93 ± 8.36	100 ± 0	100 ± 0

Considering the significant activity demonstrated by the acetone and ethanolic extracts, the assay protocol was modified to introduce a second dose of plant extract at the same concentration on the fifth day post infection. Significant inhibition of bacterial growth was observed in all 7 strains after the double stimulus. Both the extracts also exhibited increased percent inhibition (≥ 90%) in the intracellular assays with the double stimulus (Table 3).

A higher concentration (100 μg/ml) of the plant extracts (acetone and ethanolic) was also tested and showed augmented bacterial kill in all 7 strains (Table 3).

### Axenic assay under hypoxic conditions

**Figure 2:**
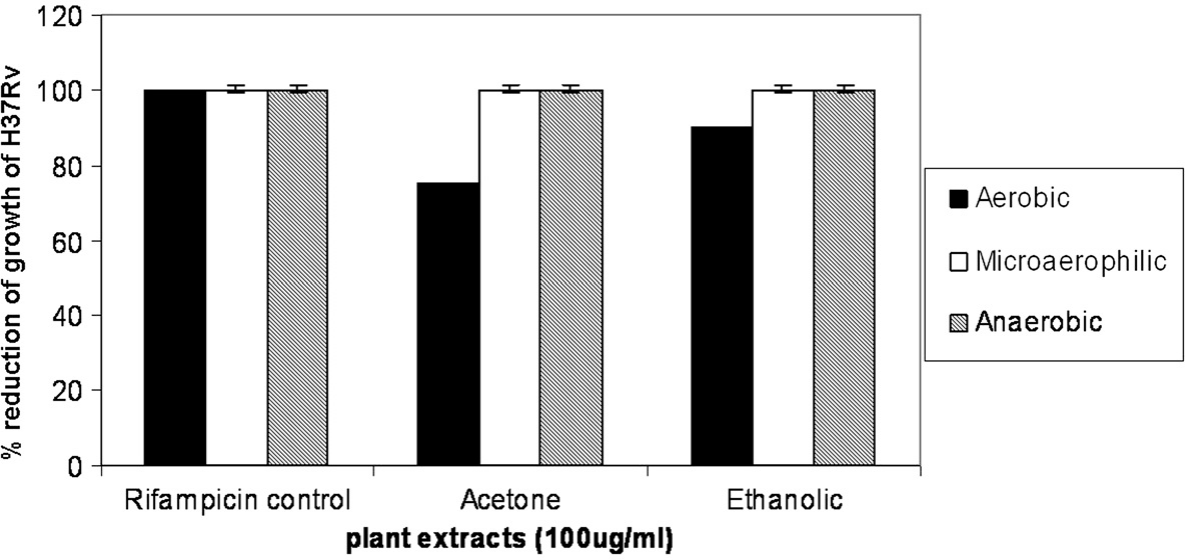
**Effect of the *Alpinia galanga *extracts on H37Rv growth under hypoxic conditions.** The figure represents percent reduction in growth of H37Rv by acetone and ethanolic extracts of *A. galanga* under reducing oxygen concentration. Rifampicin was used as a positive control for inhibition of growth.

The activity of the plant extracts was tested under reduced oxygen concentrations to mimic conditions for studying the effect of plant extracts on latent bacteria and to dissect the intracellular environment. Hence the assay was restricted to H37Rv only. Significant inhibition of the bacteria was observed with acetone and ethanolic extracts when compared to the 1:100 VMTB growth control (Figure 2).

## Discussion

There has been no anti-TB drug introduced in the past 30 years and the rapid acquisition of drug resistance to the existing drugs necessitates development of new, effective and affordable anti-TB drugs [24]. Plant-derived antimycobacterial compounds belong to an exceptionally wide diversity of classes, including terpenoids, alkaloids, peptides, phenolics and coumarins. Hence medicinal plants remain an important resource to find new therapeutic agents [25]. The advantages of using antimicrobial compounds from medicinal plants include fewer side effects, better patient acceptance due to long history of use, reduced costs and cultivability rendering them renewable in nature [26].

*A. galanga* is a known wide spectrum antibacterial agent. Soundhari and Rajarajan [16] have reported the antimycobacterial activity of galangal against isoniazid resistant isolates at minimum inhibitory concentration (MIC) of 250 μg/ml. In our study, ethanolic extract of *A. galanga* was found to be bactericidal to M.tb under axenic aerobic conditions at 50-100 μg/ml. The variation in the active concentrations could be due to differences in the method of extraction and the assay used. The antimycobacterial activity of the essential oils from *A. galanga* has been reported since 1957 [27]. The phenolic components of the oils have been reported to act as membrane permeabilisers [28]. In addition, it has been suggested that low oxygen levels can enhance the activity of essential oils. Diminished oxygen supply leads to fewer oxidative changes in the essential oils and/or that cells obtaining energy via anaerobic metabolism are more sensitive to the toxic action of essential oils [28]. The enhanced activity of the extracts under anaerobic conditions could thus be attributed to this. The ability of the *A. galanga* extracts to remain active under hypoxic conditions could further be explored for the treatment of LTBI, where the bacteria are in a non-replicating and dormant state and remain unaffected by the conventional TB antibiotics.

Latha et al. [29] have demonstrated the plasmid curing based activity of crude acetone extract of *A. galanga*. The principal compound responsible for the activity was identified as 1´ acetoxychavicol acetate. The activity of this compound offers new perspectives to control the replication and thus exhibits its potential to disrupt plasmid replication and re-sensitize bacteria to antibiotics. Though M.tb may not contain plasmids, it is possible that 1´ acetoxychavicol acetate (also detected in the extracts used in the study) may act on the genome of M.tb to render the bacterium sensitive to antibiotics. Antimicrobials from plants have been found to be enhancers in that though they may not have any antimicrobial properties alone, but when consumed in tandem with conventional drugs they may enhance the effect of that drug [30]. Studies on synergism between known antimicrobial agents and bioactive plant extracts have also been demonstrated [31-33].

The acetone and ethanolic extracts were found to show antimycobacterial activity under intracellular conditions. It appears that the *A. galanga* extracts are able to penetrate into the cells (A549) and act on the bacterium residing intracellularly, unlike the action of some antibiotics viz gentamycin which cannot penetrate the cells and exert their action [34].

## Conclusions

In conclusion, the antimycobacterial activity of *A. galanga* observed under aerobic and anaerobic axenic conditions and in the intracellular assay system could be due to different phytoactive components acting with varied mode of action(s). Furthermore, in depth studies to determine the active component(s) could lead to potential formulations that serve not only as adjunct to current therapy but as options in emerging clinical drug resistance.

The use of *A. galanga* in diet and traditional medicines has been extensively reported, its regular intake within feasible limits could act as an adjunct to the ongoing conventional TB therapy. Additionally the promising activity of *A. galanga* under microaerophilic and anaerobic conditions could also be developed further as a treatment for latent infection in TB-endemic regions where more than one third of the population act as reservoirs of dormant/non replicating M.tb [35].

Athough the results from the present study are indicative that *A. galanga* has promising antimycobacterial activity, studies using more isolates/strains of M.tb are needed.

## Competing interest

The authors declare that they have no competing interests.

## Authors’ contributions

PG and PB have conducted the intracellular and axenic anaerobic assays, analyzed the results and drafted the manuscript. DD has conducted the MABA and edited the manuscript. MT has assisted in the MABA and edited the manuscript. PD has undertaken the phytochemistry of the plant extracts. PT has sourced, cultivated and authenticated the plant material. TB is responsible for the study. All authors read and approved the final manuscript.

## Pre-publication history

The pre-publication history for this paper can be accessed here:

http://www.biomedcentral.com/1472-6882/14/84/prepub
